# Cd^2+^ + Cr^6+^ causes toxic effects on chromosomal development of microspore in *Carthamus tinctorius*

**DOI:** 10.3934/genet.2019.1.1

**Published:** 2019-04-10

**Authors:** Neha Mittal, Anand kumar Srivastava

**Affiliations:** Department of Botany C.C.S. University Meerut-250004, India

**Keywords:** *Carthamus tinctorius*, Cd^2+^ + Cr^6+^, Meiotic changes, intra-category hybrids, chromosomal abnormalities, pollen grains

## Abstract

Intra-category hybrids of *Carthamus tinctorius* were analyzed for the genetic toxicity in detail for effect of Cd^2+^ + Cr^6+^ on reproductive biology of *Carthamus tinctorius*. Five partially tolerant and five non-tolerant accessions of *Carthamus tinctorius* after screening were crossed to produce intra-category hybrid. These two heavy metals in combination influenced antagonistically first as well as second meiotic divisions inducing various kinds of anomalies and reduced the number of pollen grains per anther and significantly increased pollen sterility. A differential response for the amount of meiotic irregularity was recorded between different treated sets of hybrids in (Cd + Cr) treated sets. This could be due to differential response of the genotypes for the same concentration of (Cd + Cr). These two heavy metals in combination reduced the number of pollen grains per anther and significantly increased pollen sterility.

## 1. Introduction

It will not be wrong to say that industrial progress has become synonymous with an assault on nature. We only think about the betterment of our race and develop ‘norms’ which may help us in getting maximum and non-exhaustible natural resources at the cost of polluting our own environment. Most of the industries release a huge amount of heavy metal and contaminated the soil and posed a serious issue to sustainable agriculture and human health worldwide. Almost all of them are dangerous to human health and to the plants. Cadmium contamination in soil has posed a serious issue to sustainable agriculture and human health worldwide. Exposure to cadmium can occur in the workplace or from contaminated food stuff and can result in emphysema, renal failure, cardiovascular disease and perhaps cancer. Chromium, particularly hexavalent chromium, has been designated as a priority pollutant due to its ability to cause mutation and cancer. The two largest sources of chromium emission in the atmosphere are the chemical manufacturing industries and combustion of natural gas, oil and coal. Assessment of the genetic impact of heavy metal pollution on plants is of great importance as plants play a major role to maintain ecological balance in nature and fulfill our various requirements. Moreover, plants are used as biosensors of genetic toxicity of heavy metal pollutants. In this research, various meiotic anomalies were considered as a biomarker of genetic toxicity. The present paper discusses the toxic impacts of Cd^2+^ + Cr^6+^ on reproductive biology of some accessions of *Carthamus tinctorius*, an important oil crop.

## 2. Materials and methods

Cadmium chloride (CdCl_2_) and sodium dichromate (Na_2_Cr_2_O_7_) were used as sources of Cd^2+^ and Cr^6+^ respectively. Partially tolerant (PT) and non-tolerant (NT)* accessions were crossed with each other to produce ‘intra-category hybrids’. Treatment with 10^−3^ M of Cd^2+^ + Cr^6+^ solution was given to intra-categiry hybrids of *Carthamus tinctorius* (Table 1). For this twigs taken from nine weeks old plants were hydroponically cultured in 10^−3^ M of Cd^2+^ + Cr^6+^ prepared in Hoagland's solution. Control sets were raised in Hoagland's solution.

**Table 1.** List of accessions of *Carthamus tinctorius.*

Hybrid Lab Code	Accession	Hybrid Lab Code	Accession
NC-1	T-61XT-128	NC-6	T-128XT-157
NC-2	T-61XT-141	NC-7	T-128XT-235
NC-3	T-61XT-157	NC-8	T-141XT-157
NC-4	T-61XT-235	NC-9	T-141XT-235
NC-5	T-128XT-141	NC-10	T-157XT-235

Young floral buds of safflower were fixed for 48 hours in freshly prepared Cornoy's fluid II (absolute alcohol: chloroform: glacial acetic acid: 6:3:1) having a pinch of ferric chloride. These samples were then stored in 70% ethanol in refrigerator. Anthers of appropriate size were smeared and squashed, one at a time, in 1.5% acetocarmine. All the studies were made from temporary un-squashed as well as squashed preparations. Different parameters were used to determine the effect of Cd^2+^ + Cr^6+^ on male meiosis and fertility, the parameters are as follows:

(1) Chromosome configuration at diakinesis including chiasmata frequency
(2) Types and frequency of meiotic anomalies
(3) Pollen diameter (µm)
(4) Pollen fertility
(5) Number of pollen per anther

$$Xta/PMC=\frac{Total\;number\;of\;Xta\;in\;analyzed\;PMCs}{Total\;number\;of\;PMCs\;analyzed}\times 100$$ (1)

$$Frequency\;of\;an\;anomalous\;stage=\frac{Numbr\;of\;PMCs\;showing\;an\;anomaly}{Total\;number\;of\;PMCs\;at\;that\;meiotic\;stage}\times 100$$ (2)

$$Total\;meiotic\;anomalies=\frac{Number\;of\;PMCs\;showing\;anomalies}{Total\;number\;of\;PMCs}\times 100$$ (3)

$$Response\;coefficient\;\left(RC\right)=\frac{VT-VC}{VC}$$ (4)

Where VT = value of the treated set; VC = value of the control set. (The concentration to be used for quantification and tolerance, 235 accessions for Cd^2+^ + Cr^6+^ was estimated using experimental designs that could not be given here.)

## 3. Observation

Data for RCs related to the effect of Cd^2+^ + Cr^6+^ on the chromosome configuration at diakinesis including chiasmata frequency are presented in Table 2.

**Table 2.** Chromosome configurations at metaphase I in control and treated accessions.

Acc.	Para.	Ring Bivalents			Chaismata/PMC		
		Mean ± SE	Range	RC	Mean ± SE	Range	RC
NC-1	C	7.93 ± 0.23	6.00–9.00	0.39	19.93 ± 0.23	18.00–21.00	0.16
	T	4.80 ± 0.14	4.00–6.00		16.80 ± 0.14	16.00–18.00
NC-2	C	7.80 ± 0.11	7.00–8.00	0.40	19.80 ± 0.11	19.00–20.00	0.16
	T	4.67 ± 0.19	4.00–6.00		16.67 ± 0.19	16.00–18.00
NC-3	C	8.60 ± 0.25	7.00–10.00	0.50	20.60 ± 0.25	19.00–22.00	0.21
	T	4.33 ± 0.19	3.00–5.00		16.33 ± 0.19	15.00–17.00	
NC-4	C	8.00 ± 0.20	7.00–9.00	0.48	20.00 ± 0.20	19.00–21.00	0.19
	T	4.13 ± 0.24	3.00–6.00		16.13 ± 0.24	15.00–18.00
NC-5	C	7.73 ± 0.15	6.00–8.00	0.43	19.73 ± 0.15	18.00–20.00	0.17
	T	4.40 ± 0.49	2.00–8.00		16.40 ± 0.49	14.00–20.00	
NC-6	C	7.20 ± 0.20	6.00–8.00	0.44	19.27 ± 0.23	18.00–21.00	0.17
	T	4.00 ± 0.17	3.00–5.00		16.00 ± 0.17	15.00–17.00
NC-7	C	7.40 ± 0.13	7.00–8.00	0.35	19.40 ± 0.13	19.00–20.00	0.12
	T	4.80 ± 0.24	4.00–7.00		17.07 ± 0.37	16.00–20.00	
NC-8	C	8.60 ± 0.16	8.00–10.00	0.51	20.60 ± 0.16	20.00–22.00	0.21
	T	4.20 ± 0.17	3.00–5.00		16.20 ± 0.17	15.00–17.00
NC-9	C	8.20 ± 0.22	6.00–9.00	0.59	20.20 ± 0.22	18.00–21.00	0.23
	T	3.33 ± 0.27	2.00–5.00		15.47 ± 0.34	14.00–18.00	
NC-10	C	8.27 ± 0.25	7.00–10.00	0.48	20.27 ± 0.25	19.00–22.00	0.20
	T	4.27 ± 0.28	3.00–6.00		16.27 ± 0.28	15.00–18.00

**Figure 1.**
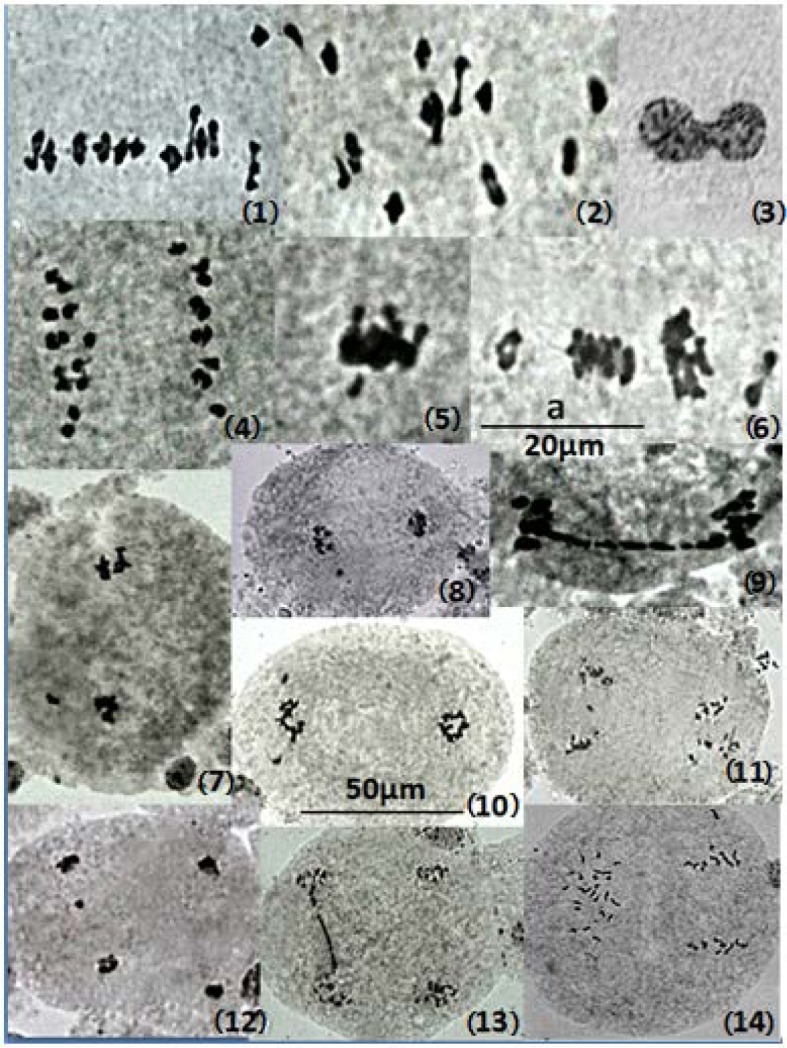
(1) Retarded movement of bivalents for metaphase I alignment, (2) Precocius disjunction of bivalents at metaphase I, (3) Restitution nucleus during prophase I, (4) Unequal distribution of chromosomes at anaphase I, (5) Clumping of bivalents at metaphase I, (6) Grouping of bivalents at metaphase I, (7) Retarded movement of chromosomes at metaphase II, (8) Micronuclei at telophase I, (9) Chromatin bridge at anaphase I, (10) Precocious disjunction of chromosome at metaphase II, (11) Laggard at anaphase II, (12) Micronuclei at telophase II, (13) Chromatin bridge at anaphase II, (14) Asynchrony within cell. (Bar ‘a’ = 20µm for Figure 1(1)–(6) and (9), bar ‘b’ = 50µm for Figure 1(7), (8), (10)–(14)).

The control sets very often possessed pollen mother cells (PMCs) with twelve ring bivalents that were drastically reduced in treated sets. Number of ring bivalents at diakinesis decreases up to only two out of twelve in hybrid NC-5 and NC-9. Consequently, in intra-category hybrids, frequency of chiasmata per PMC reduced significantly in response to Cd^2+^ + Cr^6+^ treatment. Reduction in frequency of chiasmata will decrease the chance of recombination which plays important role in genetic variability. Control plants of intra-category hybrids accessions of safflower had more or less normal meiosis while treatment with Cd^2+^ + Cr^6+^ significantly increased the frequency of PMCs with meiotic irregularities. Frequencies of meiotic anomalies increased significantly in intra-category hybrid plants in comparison to control sets. Several type of meiotic irregularities related to spindle organization and function, chromatin agglutination and chromosome condensation like, retarded movement of bivalents for metaphase I alignment (Figure 1(1)), precocious disjunction of bivalents at metaphase I (Figure 1(2)), Restitution nucleus during prophase I (Figure 1(3)), Unequal distribution of chromosomes at anaphase I (Figure 1(4)), clumping of bivalents at metaphase I (Figure 1(5)), Grouping of bivalents at metaphase I (Figure 1(6)), Retarded movement of chromosomes at metaphase II (Figure 1(7)), micronuclei at telophase I (Figure 1(8)), and chromatin bridge at anaphase I (Figure 1(9)). precocious disjunction of chromosome at metaphase II (Figure 1(10)), laggard at anaphase II (Figure 1(11)), micronuclei at telophase II (Figure 1(12)), chromatin bridge at anaphase II (Figure 1(13)), and asynchrony within cell (Figure 1(14)) were also induced by the Cd^2+^ + Cr^6+^ treatment. Data related to frequency of meiotic anomalies are presented in Table 3. Data given in Table 3 revealed that the frequency of asynchrony in telophase II was maximum upto 33.33% in NC-7, while frequency of restitution nuclei in prophase I was least amongst the various types of anomalies ranging from 9.38 to 12.90. Late movement and precocious disjunction was found to be more common in metaphase I. Frequency of late movement was maximum upto 31.43% in hybrid NC-7 while frequency of precocious disjunction was maximum up to 32.31% in NC-7.

Laggards were most common in anaphase I up to 30.71% in hybrid NC-4, while Chromatin bridge was least up to 9.84% in hybrid NC-8. Frequency of micronuclei in telophase I is maximum up to 17.54% in hybrid NC-9. Frequency of precocious disjunction in metaphase II was maximum up to 321.81% in hybrid NC-7, while maximum frequency of late movement and clumping in metaphase II was 30.23% (NC-7) and 28.68% (NC-3) respectively. Laggards were found to be most common in anaphase II with maximum frequency up to 29.66 in hybrid NC-7, while frequency of chromatin bridge was maximum up to 14.81% in hybrid NC-10. Asynchrony was most common in telophase II upto 33.33% in hybrid NC-7, while frequency of micronuclei was maximum up to 19.33% in hybrid NC-10.

In treated set number of pollen per anther and diameter of pollen reduce significantly in comparison to control set. Significant increase in pollen sterility was another Cd^2+^ + Cr^6+^ induced adverse effect. Data for RCs related to the effect of Cd^2+^ + Cr^6+^ on number of pollen per anther, diameter of pollen and pollen sterility are presented in Table 4. Frequncye of pollen sterility was maximum up to 42.24% in hybrid NC-9, while minimum frequency (32.17%) was found in hybrid NC-8. Minimum value of RC (−0.52) in hybrid NC-1revealed the maximum decrease in number of pollen per anther while RC value (−0.31) in hybrid NC-10 showed the minimal decrease in number of pollen per anther. RC value (−0.53) in hybrid NC-1 showed the maximum decrease in size of pollens while RC value (−0.24) in hybrid NC-6 showed the minimal decrease in size of pollen.

**Table 3.** Frequency (%) of different meiotic anomalies in safflower induced by Cd^+2^ + Cr^+6^ treatment.

Anomalies	NC-1		NC-2		NC-3		NC-4		NC-4		NC-6		NC-7		NC-8		NC-9		NC-10	
	F_1_		F_1_		F_1_		F_1_		F_1_		F_1_		F_1_		F_1_		F_1_		F_1_	
	Co	Tr	Co	Tr	Co	Tr	Co	Tr	Co	Tr	Co	Tr	Co	Tr	Co	Tr	Co	Tr	Co	Tr
MEIOSIS I																				
Prophase I																				
Restitution nucleus	-	12.90	-	-	-	9.38	-	-	-	-	-	12.50	-	11.70	-	10.89	-	8.33	-	-
Metaphase I																				
Late movement	-	26.98	6.42	28.91	5.21	28.46	-	24.55	5.50	32.12	7.95	27.54	8.99	31.43	9.41	25.60	-	30.77	8.14	25.00
Precocious disjunction	4.90	24.60	-	26.13	-	27.74	9.18	24.60	3.81	26.62	8.74	22.96	7.06	32.31	-	21.84	7.84	27.66	6.52	30.00
Clumping	6.19	27.14	6.67	28.10	10.68	28.91	-	26.36	-	30.08	-	24.62	-	29.10	5.88	24.27	4.95	30.71	-	28.80
>1 group	2.70	28.68	-	25.69	-	25.95	6.32	23.93	3.16	28.70	4.65	30.43	3.57	31.08	-	18.75	2.20	26.67	-	24.81
Anaphase I																				
Lagging	3.81	27.13	-	25.47	3.19	25.81	4.30	30.71	-	25.44	3.09	25.21	4.27	24.81	5.66	22.22	5.05	20.49	-	26.23
Chromatin bridge	-	14.02	-	13.13	-	14.81	-	11.54	-	16.83	-	9.86	-	13.45	-	9.84	-	13.51	-	14.78
Unequal distribution	-	12.50	-	13.59	-	12.75	-	13.59	-	10.31	-	14.81	-	12.12	-	14.43	-	12.24	-	14.02
Telophase I																				
Micronuclei	-	14.02	-	15.63	-	13.86	-	16.82	2.02	17.17	-	14.04	-	13.85	1.90	15.60	-	17.54	-	15.18
Metaphase II																				
Late movement	5.21	23.97	6.32	27.19	-	27.64	6.59	26.96	-	29.77	5.21	23.31	-	30.23	7.23	19.67	7.53	28.47	7.55	28.47
Precocious disjunction	-	22.05	5.21	24.79	4.21	26.98	5.21	29.37	4.08	27.66	-	26.57	5.50	32.81	-	25.81	-	31.54	6.52	25.44
Clumping	5.10	27.91	2.25	27.59	4.30	28.68	-	21.01	6.60	28.07	3.64	22.22	-	26.32	5.41	22.58	4.44	25.56	-	28.23
Anaphase II																				
Lagging	-	20.18	4.65	23.85	-	22.69	-	19.75	2.33	21.02	3.19	22.31	-	29.66	5.10	21.43	-	28.83	5.07	25.83
Chromatin bridge	-	10.68	-	9.18	-	12.77	-	13.54	-	12.82	-	14.68	-	13.89	-	12.50	-	13.73	-	14.81
Telophase II																				
Micronuclei	-	12.50	1.19	12.90	-	14.02	2.11	16.38	-	15.53	-	14.02	-	16.07	-	13.82	-	17.65	-	19.33
Asynchrony	6.67	27.59	9.38	32.85	11.54	30.60	7.84	28.57	8.85	32.09	7.92	27.15	11.50	33.33	8.57	22.40	7.89	31.91	11.54	29.58

**Table 4.** Cd^+2^ + Cr^+6^ induced changes in pollen sterility, pollen/anther and pollen diameter (µm).

Acc.	Pollen Sterility			Pollen/Anther			Pollen Diameter		
	Para.	%PS	RC	Mean ± SE	Range	RC	Mean ± SE	Range	RC
NC-1	Con.	1.13		1044.80 ± 18.57	952.00–1132.00		47.70 ± 1.22	40.00–52.00	
	Tre.	38.06	32.69	500.00 ± 9.91	464.00–548.00	−0.52	22.55 ± 0.75	17.00–27.00	−0.53
NC-2	Con.	1.73		969.60 ± 25.34	856.00–1052.00		47.75 ± 0.58	45.00–54.00	
	Tre.	34.67	18.99	630.40 ± 26.05	540.00–832.00	−0.35	26.20 ± 0.35	24.00–28.00	−0.45
NC-3	Con.	1.01		1121.60 ± 18.85	1032.00–1196.00		47.85 ± 0.55	45.00–52.00	
	Tre.	36.11	34.93	574.40 ± 18.50	476.00–672.00	−0.49	32.35 ± 0.52	30.00–39.00	−0.32
NC-4	Con.	0.98		1081.20 ± 10.78	1032.00–1136.00		46.70 ± 0.95	42.00–54.00	
	Tre.	37.74	37.68	634.80 ± 14.12	568.00–732.00	−0.41	23.80 ± 0.75	20.00–28.00	−0.49
NC-5	Con.	0.87		1115.60 ± 23.84	956.00–1208.00		47.60 ± 0.47	43.00–51.00	
	Tre.	35.67	40.02	701.20 ± 11.11	648.00–756.00	−0.37	25.90 ± 1.13	18.00–34.00	−0.46
NC-6	Con.	1.04		1158.00 ± 12.67	1092.00–1216.00		45.85 ± 0.64	43.00–52.00	
	Tre.	38.24	35.71	677.20 ± 20.11	596.00–768.00	−0.42	34.85 ± 1.03	26.00–38.00	−0.24
NC-7	Con.	0.99		1132.80 ± 26.90	976.00–1284.00		47.75 ± 0.54	45.00–52.00	
	Tre.	34.09	33.6	666.40 ± 40.16	476.00–844.00	−0.41	29.45 ± 0.98	24.00–39.00	−0.38
NC-8	Con.	0.56		1094.00 ± 22.89	992.00–1192.00		46.65 ± 0.60	44.00–50.00	
	Tre.	32.17	56.26	728.00 ± 26.34	624.00–904.00	−0.33	29.75 ± 0.41	29.00–34.00	−0.36
NC-9	Con.	1.03		1094.40 ± 27.43	892.00–1208.00		50.25 ± 0.81	46.00–59.00	
	Tre.	42.24	39.97	640.40 ± 19.64	544.00–724.00	−0.41	25.60 ± 0.80	22.00–32.00	−0.49
NC-10	Con.	1.09		1076.00 ± 16.92	1016.00–1192.00		47.00 ± 0.36	45.00–49.00	
	Tre.	35.17	31.18	738.40 ± 23.40	644.00–864.00	−0.31	30.50 ± 0.34	29.00–32.00	−0.35

## 4. Discussions

Sexual reproduction has probably been favored by evolution because the random recombination of genetic information improves the chance of producing at least some offspring that will survive in an unpredictably variable environment. Sexual life cycles produce genetic variation among offspring. The process of meiosis and fertilization are the unique trademarks of sexual reproduction. The biochemical processes underpinning meiosis are highly conserved but can be influenced by both abiotic and biotic stresses [1]. Treatment of (Cd + Cr) in safflower induced various meiotic anomalies related to chromatin agglutination, chromosome condensation and spindle anomalies. A differential response for the amount of meiotic irregularity was recorded between different treated sets of intra-category hybrids in (Cd + Cr) treated sets. This could be due to differential response of the genotypes for the same concentration of (Cd + Cr). Somashekhar [2] analysed induced meiotic abnormalities in *Chlorophytum* *amaniense*, by treating the floral buds directly with water contaminated with the effluent of a dye industry. Ramel and Engstrom [3] conducted preliminary investigations on the effect of an organic mercurial (methyl mercury hydroxide) on microsporogenesis of *Tradescantia*, reporting the induction of spindle fibre inactivation.

A significant decrease in frequency of ring bivalents were reported due to treatment of (Cd + Cr), therefor reducing X-ta/PMC to a significant extent. Similar results were reported by Sharma, Tomar and Madaan [4]–[6] in several crop species treated with different heavy metals and other water pollutants. Chiasmata are the cytological manifestation of genetic crossing over; therefore their reduction could be interpreted as reduction in recombination frequency with consequent decline in genetic variability. Complete breakdown of spindle organization resulted in the formation of restitution nuclei, while the presence of “longitudinal non-functional” zones caused the congregation of chromosomes in more than one groups. Observed restitution nuclei chiefly resulted due to chromatin agglutination [7].

The presence of retarded movement of chromosome for metaphase alignment and lagging of chromosome evident that (Cd + Cr) could induce functional anomalies in spindle apparatus. Bivalents found clumped in single or different groups at metaphase I/II due to depolymerization of nucleic acids or partial dissociation of nucleoproteins and alteration in their pattern of organization [8]. Sticky chromosome represent poisoned chromosome with sticky surface and probably lead to cell death [9]–[10]. The induction of lagging could be attributed to the failure of the normal organization and function of the spindle apparatus. Such type of abnormalities is due to the loss of microtubule of spindle fibers. Lagging of chromosome during anaphase may be the consequence of several cytological anomalies (1) Late movement of chromosome for metaphase alignment (2) Inhibition of attachment on metaphase of centric chromosome (3) Absence of centromere (4) Late disjunction of sister centromeres (5) Localized stickiness in centromeric portions of chromosomes, and (6) Partial inactivation of force generating system responsible for chromosome displacement. Induction of lagging, reported presently appears due to all above-mentioned reasons. Meiotic bridge may arise due stickiness in the localized portions of chromosome appears a local reason for the formation of bridges due to induced retardation to chromatin disjunction. Chromatin bridge may arise due stickiness in the localized portions of chromosome appears a local reason for the formation of bridges due to induced retardation to chromatin disjunction. Formation of bridges at anaphase indicates of exchange between the chromosomes taking part in exchange [11]–[13]. The formation of bridges could be attributed to chromosome stickiness and to chromosome breakage and reunion [14]. The anaphasic bridges obtained might be due to structural changes to deficiency and translocation type, some of them surviving until the late telophase, indicative of their stability [15]. Rai and Kumar [16] reported the stickiness, precocious movement, laggards, non-synchronous division, forward movement, unequal separation, bridges, and cytoplasmic channel in meiosis of treated sets of all the inbred lines of maize.

Gaulden [17] postulated that sticky chromosomes may result from the defective functioning of one or two types of specific non-histone proteins involved in chromosome organization which are needed for chromatid separation and segregation. The altered functions of these proteins leading to stickiness may be caused by mutations in the structural genes coding for them (hereditary stickiness) or by the action of mutagens on the proteins (induced stickiness). According to Kumar and Rai [18], it seems most probable that the heavy metals may have caused some kinds of gene mutations which led to incorrect coding of some non-histone proteins involved in chromosome organization. Chromosome stickiness leading to sticky metaphase and precocious separation of chromosome is possibly due to chemical breaking the protein moiety nucleoprotein backbone [19]. It seems most probable that interaction of Cadmium with chromatin proteins leads to incorrect coding of some non-histone proteins involved in chromosome organization, ultimately resulting in cytogenetic abnormalities [20]. The occurrence of micronuclei is regarded as reliable parameter for clastogenecity or mutagenicity of an agent [21],[22]. Therefore, presence of these anomalies is also confirming the toxic effects of (Cd + Cr) on spindle components

## 5. Conclusions

Production of >4 spores per PMC was due to the appearance of >4 nuclei in a PMC because of micronuclei formation. (Cd + Cr) treatement caused significant decrease in the number of pollen grains per anther. The reason for the decline in the number of pollen grains could be either lowering of the number of PMCs per anther or the arrest of meiosis yielding lesser number of spores per PMC. Significant increase in pollen sterility was another adverse effect. The reason for this could be induction of meiotic abnormalities as well as induction of gene mutations influencing development and maturation of pollen grains after meiosis.
